# Designing a Smartphone-Based Virtual Reality App for Relaxation: Qualitative Crossover Study

**DOI:** 10.2196/62663

**Published:** 2025-02-13

**Authors:** Amandine Verstegen, Tom Van Daele, Bert Bonroy, Glen Debard, Romy Sels, Marlon van Loo, Sylvie Bernaerts

**Affiliations:** 1 Psychology and technology Centre of Expertise - Care and Well-being Thomas More University of Applied Sciences Antwerp Belgium; 2 Mobilab & Care Centre of Expertise - Care and Well-being Thomas More University of Applied Sciences Geel Belgium; 3 Prevention and empowerment Centre of Expertise - Care and Well-being Thomas More University of Applied Sciences Antwerp Belgium

**Keywords:** smartphone-based virtual reality, virtual reality, relaxation, stress, user experience, mobile phone

## Abstract

**Background:**

Accumulating evidence supports the use of virtual reality (VR) in mental health care, with one potential application being its use to assist individuals with relaxation exercises. Despite studies finding support for the potential of VR to effectively aid in relaxation, its implementation remains limited outside of specialized clinics. Known barriers are insufficient knowledge regarding VR operation, lack of availability of VR relaxation apps tailored to local health care systems, and cost concerns. Unfortunately, many VR relaxation apps are designed exclusively for stand-alone headsets, limiting accessibility for a broad audience.

**Objective:**

We aimed to design an accessible, smartphone-based VR relaxation app based on user preferences. This paper describes the assessment of 2 stand-alone VR relaxation apps and the resulting smartphone-based VR relaxation app design.

**Methods:**

Overall, 30 participants (n=23, 77% women; n=7, 23% men) took part in 2 separate VR sessions, assessing 1 of the 2 VR relaxation apps (Flowborne and Calm Place) in each session. After each session, participants were presented with open-ended questions to assess their experiences via a web-based survey tool. These questions explored positive and negative features, shortcomings, and suggestions for improvements while also allowing space for additional remarks concerning the 2 VR relaxation apps. Three of the authors analyzed the responses using inductive thematic analysis, a process comprising 6 phases.

**Results:**

Across both the apps, 5 recurring themes and 13 recurring subthemes were identified in the participants’ answers: audio (music and sounds, guidance), visuals (content, realism, variation and dynamics in the environment), features (language, options, feedback and instructions, duration, exercise), implementation (technical aspects, cybersickness, acceptability and usability), and experience. We analyzed the participants’ findings and conducted a literature review, which served as the basis for developing the app. The resulting app is a Dutch-language, smartphone-based VR relaxation app, with customization options including 3 types of relaxation exercises, 2 guiding voices, and 3 different environments. Efforts have been made to ensure maximum variation and dynamism in the environments. Calming music and nature sounds accompany the exercises. The efficacy and effectiveness of the resulting app design were not assessed.

**Conclusions:**

This study provides insights into key features of VR relaxation apps, which were subsequently used for the development of a novel smartphone-based VR relaxation app. Further research concerning the effectiveness of this app, along with a broader evaluation of the efficacy and user feedback for smartphone-based VR relaxation apps, is needed. More generally, there is a clear need for more research on the impact of interactivity, biofeedback, and type of environment in VR relaxation.

## Introduction

### Background

Immersive virtual reality (VR) has a long-standing history in the mental health domain. Using computer-generated environments or 360-degree videos, patients experience being in the virtual environment, rather than the real world [1-3]. In therapy, VR boasts a clear-cut advantage by affording the therapist absolute control over the emotional intensity of the environment, thereby facilitating customization in response to the unique needs of the patient [4]. As VR becomes more accessible, it is being explored for a wide range of applications in mental health care. These include exposure therapy for patients with anxiety disorders [5] or posttraumatic stress disorder [6], distraction techniques for children to manage pain during burn wound treatment [3,7], body swapping for addressing eating and weight disorders [8], and using avatars with customized voices to treat patients experiencing auditory hallucinations [9]. In addition, VR is being explored as a tool to deliver or facilitate psychoeducation, behavioral activation, treatment for obsessive-compulsive disorder, addiction treatment, rehabilitation following traumatic brain injuries, and more [10,11].

Another potential application of VR is to help facilitate relaxation in individuals experiencing stress. VR relaxation apps typically present a computer-generated or 360-degree captured natural environment, such as a beach or forest, sometimes with accompanying auditory breathing or meditation exercises. The prevalence of natural environments in VR relaxation stems from research confirming their stress-reducing qualities [12]. Pizzoli et al [13] described 2 efficacious approaches to VR for promoting relaxation, namely, relaxing VR and engaging VR, and proposed a new approach, namely, personalized VR. Relaxing VR is characterized by visual content illustrating generic, pleasant environments, such as nature-based scenes, and auditory content based on common relaxation techniques, such as meditation and progressive muscle relaxation, with the aim of inducing control over one’s body. In contrast, engaging VR uses more specific scenarios and interactive sceneries with a focus on empowering and training the user to regulate one’s emotions [13]. Personalized VR was explored as a third, theory-based approach, focusing on an individualized design and implementation. Numerous studies have affirmed the effectiveness of VR relaxation in promoting relaxation and alleviating stress [14-19]. Recent reviews by Riches et al [20-22] also demonstrate that VR relaxation is an acceptable, feasible, and effective tool for enhancing relaxation and reducing stress across diverse populations, including the general population [20], various clinical populations [21], and in workplace settings [22].

Despite an increasing body of evidence highlighting the effectiveness of VR relaxation, its routine care implementation remains limited outside of specialized clinics [23]. Technology adoption already proves challenging in mental health care [24], but several barriers hamper the uptake of VR relaxation specifically. A first barrier is that many therapists or individuals experiencing stress frequently lack the knowledge of how to operate VR [25]. Furthermore, numerous VR relaxation apps are not adapted to or available in patients’ native language. Another challenge is the fact that VR remains relatively expensive [11,26,27]. However, concerning the latter barrier, it should be noted that VR headsets exist in different price ranges. One distinction that can be drawn among VR headsets that rely on smartphones and those that can be used stand-alone. Smartphone-based VR headsets, as implied by their name, require a smartphone to function, whereas stand-alone VR headsets operate independently. The latter are frequently more sophisticated, although considerably more expensive than smartphone-based VR headsets. A smartphone-based VR headset is readily available with a starting price as low as €15 (US $15). Unfortunately, most relaxation apps are designed exclusively for stand-alone headsets, limiting accessibility for a broad audience. Notably, there is a third category, of computer-connected (or tethered) headsets. However, these are even more expensive than stand-alone VR headsets, especially because they rely on an additional powerful computer.

### Objectives

The purpose of this study is to develop a relaxation app specifically for smartphone-based VR headsets. Such VR headsets allow people to use their own smartphone, making them a potentially cheaper and more accessible alternative to stand-alone VR headsets. However, it is crucial to acknowledge that owning a smartphone is a prerequisite for using a smartphone-based VR headset. Fortunately, most of the population already owns a smartphone, making this cost less of a barrier. To overcome the previously highlighted barrier of inadequate adaptation to the local health care sector and language, the app is tailored to the local (ie, Flemish) context and developed in the Dutch language.

## Methods

### Overview

This study is 2-fold. In the first part, we assess the user’s perspectives on existing VR relaxation apps using evidence-based techniques. In the second part, we describe the development of a smartphone VR app, considering previous user feedback and relevant literature. The validation of the resulting app design is beyond the scope of this study.

### Study Design and Procedure

A crossover study to assess users’ perceptions of 2 VR relaxation apps was conducted at Thomas More University of Applied Sciences, Belgium. Participants took part in 2 separate VR sessions, exploring 1 of the 2 VR relaxation apps per session. After each VR session, participants were presented with open-ended questions to assess their experiences via the web-based survey tool QuestionPro (QuestionPro, Inc). These questions explored positive and negative features by assessing strengths (What did you like about the app?), shortcomings (What did you like less or dislike about the app?), and suggestions for improvements (What would you improve about the app?), while also allowing space for additional remarks concerning the 2 VR relaxation apps. Before and after each session, quantitative data concerning relaxation levels, positive and negative affect, attitude toward technology, and mood were also collected. However, these data are beyond the scope of this study. Each VR session lasted approximately 5 to 10 minutes and took place on 2 different days, with at least 1 week in between. The order in which the apps were presented was randomized. Each study session lasted approximately 40 minutes.

### Ethical Considerations

The study design and consent forms were approved by the Ethical Committee of the Department of Applied Psychology at Thomas More University of Applied Sciences (ECTP2223_06). Before data collection, participants were informed about the study objectives, study design, and data processing both verbally and through an information letter. They also provided written informed consent. Participants were not compensated for their participation in the study. Data were collected, handled and stored according to the European Union General Data Protection Regulation (GDPR) and International Council for Harmonisation of Technical Requirements for Pharmaceuticals for Human Use (ICH) Guideline for Good Clinical Practice (GCP). Data were deidentified and all analyses adhered to data privacy guidelines.

### Participants

A total of 30 employees (n=23, 77% women; n=7, 23% men) from Thomas More University of Applied Sciences were recruited through a call in an internal newsletter between December 2022 and January 2023, across 2 campuses in Antwerp and Geel, Belgium. The call provided information about the study objectives and study procedures. Interested employees were asked to contact the researchers via email for additional information and to schedule the data collection. Their average age was 37.17 (SD 8.91) years. Individuals with neurological conditions, such as epilepsy, or severe neck problems were excluded. There were no dropouts.

### Intervention

#### Overview

Participants were presented with 2 freely available VR relaxation apps via the Meta Quest 2 stand-alone VR headset (Meta Platforms, Inc), Flowborne (Flowborne) [28] and Calm Place (Mimerse) [23]. It is important to note that Calm Place is not publicly available, but the creators gave permission to use the app for research purposes.

#### Flowborne

Flowborne is a freely available respiratory biofeedback VR app designed to promote diaphragmatic breathing in a playful way. The app was developed in an academic context, and previous research has demonstrated its effectiveness in improving diaphragmatic breathing and breath awareness, promoting a sense of relaxation [29]. Flowborne consists of a low-polygon 3D environment, a digitally created space that uses fewer geometric shapes to ensure a smoother and faster VR experience, featuring natural elements such as grass, stones, flowers, and trees. The environments are composed of 4 different landscapes, each containing 6 different levels with an additional variation in duration (ie, short, medium, and long). The 4 different landscapes vary in terms of color themes and environmental characteristics. For instance, one of the landscapes resembles a hill, while another resembles an ocean. What makes this VR relaxation app stand out is the ability for users to navigate through the environment by breathing, accompanied by visual alterations in response to their breath (eg, grass growth and color shifts). During exercises, users are required to position the controller of the VR headset on their lower abdomen, allowing the controller to detect the expansion and contraction of the abdominal region and promote abdominal breathing. A slow, deep breathing pattern is necessary to progress fluently through the environment. There is no guiding voice throughout these exercises. At the outset, individuals have the opportunity to select a tutorial session that explains the exercise and the use of the controller on their abdomen. In addition, users experience ethereal and natural sounds during their journey in the environment. For this study, participants were instructed to complete a session of medium duration (ie, 9 minutes) with a landscape of their choice.

#### Calm Place

Calm Place is a VR relaxation app developed by Mimerse. Although this company is no longer operational, it has made its app available for research purposes upon request. In this VR relaxation app, users immerse themselves in a low-polygon natural environment, at a campfire surrounded by nature. During the exercise, one has the option to transition the environment from daytime to nighttime. The app uses a gaze-controlled interface, enabling users to navigate the environment by simply moving their heads. By directing their gaze toward specific objects, users can induce changes in the environment [23]. The exercises can be done without controllers; they are only needed to set up the exercises and customize the environment to individual preferences. This customization includes selecting whether it rains, choosing whether there are animals, setting a specific time to prevent a transition from day to night, adjusting sound volume, enabling or disabling environment sounds, choosing among 3 songs, and selecting which “relaxing object” you prefer. Exercises include guided relaxation, mindfulness exercises, or simply observing nature. In both the guided relaxation and mindfulness exercises, there is a voice that guides you step by step through the process. In the guided relaxation exercises, you can choose from different relaxation techniques: basic relaxation, muscle relaxation, and deep breathing. Similarly, in the mindfulness exercises, you can specify the session number you prefer to follow. There are also 4 different landscapes and 3 different duration options (ie, 9, 13, or 20 minutes). At the outset and conclusion, participants are asked to report on their emotional state (“How are you feeling now?”), using a scale featuring 5 emojis. For this study, participants were instructed to perform a 9-minute deep breathing exercise and were given the freedom to choose their preferred environment.

#### Data Analysis

Using inductive thematic analysis, qualitative data were analyzed by 3 team members (AV, MvL, and SB) for each app separately. The goal was to map out users’ perspectives regarding strengths, weaknesses, and potential improvements in the 2 VR relaxation apps. Although there is no singular definitive method for conducting thematic analysis, Braun and Clarke [30] have outlined a potential procedural framework consisting of 6 phases: familiarization, coding, theme development, reviewing themes, defining and naming themes, and writing the report. In the familiarization stage, the responses to the open-ended questions underwent repeated readings by each of the 3 coders individually to attain a profound understanding. Subsequently, in the second phase, a brief summary or code was provided by each of the 3 coders individually for what was said in the responses. The data were presented in Excel (version 365; Microsoft Corp) and were open-coded through semantic coding, and no predetermined codebook was used. For instance, codes such as “more realistic” were assigned to responses such as the following:

The images are very “virtual.” I think I would find it more pleasant if real images of mountains were used.

Each coder individually went through multiple iterations of coding. However, these iterations and the corresponding changes and evolution of the codes were not documented. During the third stage, the 3 coders individually searched for overarching themes and subthemes and categorized the answers to the open-ended questions using colors associated with these initial themes and subthemes. Note that, in this respect, although an inductive thematic analysis approach was adopted, deductive analysis was necessary to some extent to ensure that the codes and themes contributed to answering the research questions, which is common in thematic analysis [31]. In the fourth stage, a comparison was made among the identified themes and the data to check whether these themes constitute an accurate representation of the data and aid in addressing the research question. In this phase, coders MvL, AV, and SB compared themes and codes to reach consensus about the codes and themes and subthemes. When similar codes and themes and subthemes were present, the clearest (ie, based on consensus) terminology was withheld. When codes and themes and subthemes differed, the researchers discussed until consensus was reached. These discussions were not documented. Official names were assigned to the themes in the fifth phase, which was led by AV. These tables were subsequently checked by SB, and the theme and subtheme structure was discussed until consensus was reached. MvL was not able to partake in this stage due to practical reasons. This thematic analysis revealed that participants’ responses to the open-ended questions could be categorized according to the following themes across the 2 VR relaxation apps: audio, visuals, features, implementation, and experience. Each of the themes has been further subdivided into different subthemes. These themes and subthemes were reported in the sixth phase and translated into specific app features, with their respective findings elaborated upon in the Results section of this study.

## Results

In the first part of this section, we discuss user perspectives on existing VR relaxation apps, categorized according to the aforementioned themes and subthemes. In the second part, we describe the translation from user preferences to app features and the resulting smartphone VR app, considering this user feedback and relevant literature.

### Stage 1: Assessment of User Perspectives

Results of the thematic analyses for each separate app can be found in Tables 1 and 2. The corresponding codes and themes and translation to app features are discussed in stage 2.

**Table 1:** Summary of users’ perspectives on the Flowborne app, organized according to the themes and subthemes from the thematic analysis.

Themes and subthemes			Strengths		Areas of improvement and negative features
**Audio**					
	Music and sounds	Music found to be calming (n=14, 47%) Music mentioned spontaneously when asked for positive aspects of the app (n=9, 30%) Nature sounds (n=2, 7%)		Suggestion to add more nature sounds (n=1, 3%)	
	Guidance	Lack of guiding voice (n=3, 10%)		Preference for a guiding voice (n=1, 3%)	
**Visuals**					
	Content	Beautiful setting (n=10, 33%) Magical setting (n=1, 3%) Animals (n=4, 13%)		Desire for more animals (n=3, 10%) World feels deserted, too little life (n=1, 3%)	
	Realism	Realistic virtual setting (n=1, 3%) Magical environment (n=1, 3%)		Preference for more realistic environment (n=3, 10%) Artificial environment (n=2, 7%) Futuristic animals and elements (n=1, 3%)	
	Variation and dynamics in the environment	Appreciation for existing variety (n=2, 7%) High activity level in the environment (n=1, 3%)		Desire for more variety (n=3, 10%) Desire for more dynamism (n=2, 7%) Distraction due to environmental activity (n=1, 3%)	
**Features**					
	Language	—^a^		—	
	Options	Customizable duration (n=1, 3%)		Desire for customizable route (n=1, 3%)	
	Feedback and instructions	—		Unclear instructions and desire for example exercise (n=3, 10%) Unclear feedback at the end (n=2, 7%) Lack of reference for breathing pace (n=1, 3%) Unclear end of exercise (n=1, 3%)	
	Duration	—		Duration too short (n=2, 7%) Negative feedback on exercise duration (n=1, 3%)	
	Exercise	Movement through breathing (n=7, 23%) Visual feedback linked to breathing (n=3, 10%)		Lack of forward movement while inhaling (n=3, 10%) Tendency to exhale more to move quickly through the environment (n=1, 3%)	
**Implementation**					
	Technical aspects	—		Negative feedback on configuration of walking path (n=3, 10%) Disappointment with the configuration of the environment boundaries (n=2, 7%) Controller as a suboptimal breathing sensor (n=1, 3%) Occasional image blurriness (n=1, 3%) Occasional visual glitches (n=1, 3%)	
	Cybersickness	—		Nausea (n=2, 7%) Dizziness (n=2, 7%) Nausea and headache (n=1, 3%)	
	Acceptability and usability	Intention to use it again (n=1, 3%)		Sense of an abandoned world (n=1, 3%)	
**Experience**					
		Pleasant experience (n=5, 17%) Relaxing effect (n=5, 17%) Energizing effect (n=1, 3%) Interesting as a sleep aid (n=1, 3%) Desire to keep wearing the headset (n=1, 3%)		Competitive aspect hinders relaxation (n=1, 3%) Uncertain about repeated enjoyment (n=1, 3%) Difficulty staying awake (n=1, 3%)	

^a^None.

**Table 2:** Summary of users’ perspectives on the Calm Place app, organized according to the themes and subthemes from the thematic analysis.

Themes and subthemes		Strengths	Areas of improvement and negative features
**Audio**			
	Music and sounds	Positive feedback on nature sounds (n=9, 30%)	Desire for more nature sounds (n=2, 7%) Suggestion to add music (n=1, 3%)
	Guidance	Pleasant, relaxing voice (n=12, 40%)	Desire for continuous guiding voice counting during breathing exercise (n=4, 13%) Desire for less auditory guidance (n=3, 10%) Desire for more interaction with guiding voice (n=1, 3%) Guiding voice should not be too demanding (n=1, 3%)
**Visuals**			
	Content	Environment (n=12, 40%) Campfire (n=2, 7%) Northern lights (n=2, 7%) Animals (n=1, 3%) Beach and rocks (n=1, 3%) Vacation feeling (n=1, 3%)	—^a^
	Realism	Realistic environment (n=1, 3%)	Desire for greater realism in the environment (n=4, 13%)
	Variation and dynamics in the environment	Day-night transition (n=5, 17%) Variation in the environment (n=4, 13%) Dynamic elements (n=1, 3%) Rain (n=1, 3%)	Rain and thunder found to be unpleasant (n=5, 17%) Static environment (n=1, 3%) Monotonous environment (n=1, 3%) Desire for more dynamic elements (n=1, 3%) Desire for more variation (n=1, 3%)
**Features**			
	Language	—	Desire for the Dutch-language option to lower the threshold for certain target groups (n=3, 10%)
	Options	Ability to choose different environments (n=2, 7%) Curiosity regarding the other environments (n=2, 7%)	—
	Feedback and instructions	—	Suggestion for better preparation or a test exercise (n=1, 3%) Suggestion for instruction on appropriate posture (n=1, 3%) Suggestion for option without indicating feelings at the end (n=1, 3%)
	Duration	Satisfaction with duration (n=2, 7%) Positive feedback on customizable exercise duration (n=1, 3%)	Exercise duration found to be short (n=1, 3%)
	Exercise	—	Desire for clearer indication of breathing rhythm (n=3, 10%) Desire for visual cues for counting during breathing exercise (n=2, 7%) Discomfort expressed regarding the fixed breathing pace (n=2, 7%) Too many tasks at once (n=1, 3%)
**Implementation**			
	Technical aspects	Appreciation for minimal controller use (n=1, 3%)	Controller visible at times (n=3, 10%) Occasional visual glitches (n=3, 10%) Difficulties with controller clicking (n=1, 3%)
	Cybersickness	—	—
	Acceptability and usability	—	Distraction due to background noise (n=2, 7%) Doubts about virtual reality relaxation’s superiority over breathing exercises with only visual or video stimuli (n=1, 3%) Suggestion to use headphones (n=1, 3%) Suggestion to use the app while standing (n=1, 3%)
**Experience**			
		Pleasant experience (n=10, 33%) Relaxing effect (n=4, 13%) Easy to imagine being in such an environment (n=1, 3%) Minimal sensory stimuli (n=1, 3%)	Boring experience (n=1, 3%) Experience found to be tiring (n=1, 3%) Fear due to the dark environment (n=1, 3%)

^a^None.

### Stage 2: Translation of User Perspectives to App Features

#### Overview

**Figure 1:**
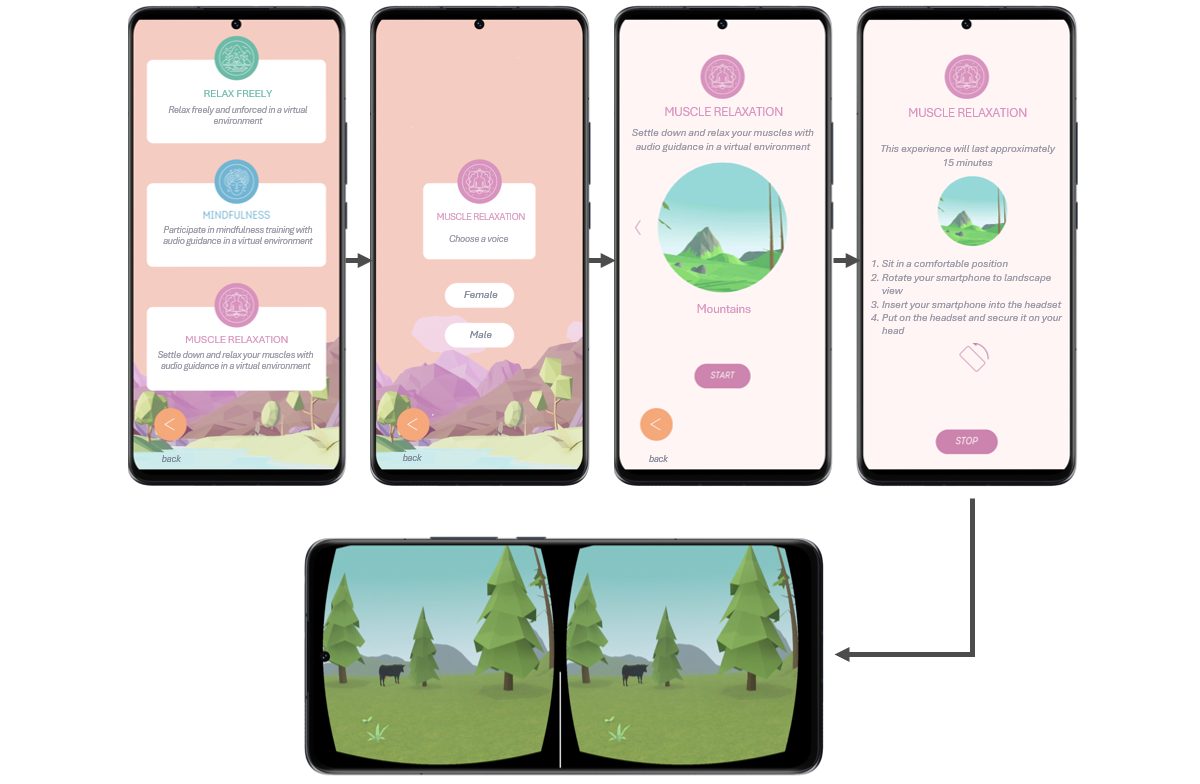
Personal configuration flow in the Immersive Mental Health app. The language in the screenshots has been translated from Dutch to English for publication purposes.

A new VR relaxation app, Immersive Mental Health (IMH; Aeroplane XR), was designed for smartphones, incorporating insights from the user assessments of the 2 stand-alone VR apps and guided by a comprehensive literature review. The app was developed by the Flemish XR developer Aeroplane XR using the Unity platform. The app’s conceptualization is presented in Figure 1. In this section, we describe the developed smartphone VR app, considering user feedback and relevant literature.

#### Audio

##### Music and Sounds

Numerous participants reported finding ethereal and nature sounds pleasant and conducive to relaxation. Studies have shown that the concurrent exposure to a nature-based VR setting and natural sounds is more effective in reducing stress compared to independent auditory or visual inputs [17,32]. Drawing upon participant evaluations and literature, we chose to integrate both natural and ethereal sounds into our new IMH app. Different nature sounds, such as chirping birds, the sound of the sea, crackling fire, wind, and others, can be heard depending on the environment chosen.

##### Guidance

Opinions on the use of a guiding voice were divided, both regarding whether it should be included at all and whether it should provide continuous guidance during breathing exercises. Some participants preferred a continuous vocal count throughout the breathing exercise, stating the following:

I would rather hear the voice continuously guiding the breath exercise with simple words like “in and out.” Counting to 10 myself kept me more focused on counting than on my breathing.

In contrast, others preferred its absence:

Hearing the guiding voice count aloud the duration of breaths made me more anxious because my pace was slower than indicated, making me afraid of not completing on time.

IMH does not require a counting voice during breathing exercises; consequently, such exercises are not included. We opted to include a guiding voice for 2 out of the 3 exercises to provide auditory explanations for the exercises, as participants have articulated a preference for clear instructions. In addition, participants were given the option to choose between a male or a female narrator. We included this customization option because the literature has consistently shown that providing personalization options leads to a higher level of relaxation [33].

#### Visuals

##### Content

A substantial number of participants found the environments to be pleasant:

Lovely environment. Feels like a vacation.

Animals and natural features such as fire and water were especially well received. Literature has also shown that natural lighting and water elements are essential for alleviating stress in VR users [34]. Grounded in the findings of this study and the evaluative study, the IMH app features natural landscapes enriched with elements such as water, fire, and animals.

##### Realism

Participants valued a realistic environment, often suggesting that the environments could be even more realistic:

The images are very “virtual.” I think it would be more pleasant if real images of mountains were used.

According to a review study, VR natural scenarios have similar effects to real nature scenarios for stress reduction [34]. However, they highlight the importance of maximizing realism in these VR scenarios to improve the immersive experience. Furthermore, research indicates that a computer-generated natural environment provides a more emotionally restorative experience compared to a 360-degree video representation of a similar setting [35]. In addition, another study found that computer-generated VR enhances the sense of presence and elevates mood more effectively than 360-degree VR [36]. Therefore, based on the literature and participant feedback, we aimed to create as realistic a computer-generated environment as possible within the limits of the processing power of a smartphone (ie, Android operated).

##### Variation and Dynamics in the Environment

The participants placed great importance on variation. They found it pleasant when there were surprising elements, and the day-night transition in Calm Place was positively assessed:

It’s nice that a day passes during these nine minutes. You see the sun first, then the stars, and then the sun again.

In addition, more participants found rain and thunder unpleasant than those who found it enjoyable. Participants also expressed a preference for dynamic elements in the environment. Consequently, we integrated these considerations into our IMH app, featuring 3 distinct natural environments, each encompassing diverse fauna, natural and dynamic elements, and day-to-night transitions.

#### Features

##### Language

Some participants believed that using an app in a language other than the user’s native language could pose a barrier. Therefore, the IMH app was developed in the Dutch language.

##### Options

Participants appreciated the option to select diverse environments and varied durations. Moreover, as noted earlier, the literature consistently demonstrates that offering customization choices results in increased levels of relaxation [33]. Therefore, we decided to incorporate a wide array of customization options. Within the IMH app, users could choose from 3 distinct environments: a tropical beach, a winter scene with the northern lights, or a mountainous landscape. Furthermore, users had the opportunity to select among 3 specific exercises: free exploration, a mindfulness exercise, or progressive muscle relaxation. In addition, participants had the option to choose between a male or female guiding voice during the mindfulness and progressive muscle relaxation exercises.

##### Feedback and Instructions

The participants expressed a preference for detailed instructions, a consideration we took into account in the development of our new IMH app. Before the exercises began, clear instructions were provided (Figure 1). First, participants were instructed to sit in a comfortable position. They were also prompted to horizontally rotate their smartphone in the direction indicated by an arrow. Subsequently, participants were asked to insert their smartphone into the VR headset. Clear instructions were also provided during the exercises: a guiding voice offered step-by-step guidance in 2 exercises. In addition, many participants indicated that they would have preferred to receive clear feedback at the end. However, the IMH app does not provide any feedback. Some participants also indicated wanting to try a sample exercise before starting the actual session:

At first, I was not sure if I was making progress. Perhaps add a small test before the meditation begins to check if you’re doing it correctly.

One of the 3 exercises in the IMH app involves simply exploring the environment without any specific tasks. This can serve as an introduction to familiarize users with the surroundings and the headset. A tutorial for the exercises is not provided in the IMH app, as the step-by-step instructions from the guiding voice eliminate the need for it.

##### Duration

During the evaluative study, certain participants expressed satisfaction with the presence of diverse duration options for the exercises. However, this feature was not integrated into the IMH app. Instead, users can exit the exercise whenever they desire. The participants also appreciated having a clear ending. Therefore, the conclusion is explicitly marked, as noted by the following statement: “your session is now complete.”

##### Exercise

Many participants found navigating the environment through breathing enjoyable in Flowborne. However, there were some remarks regarding the limitation that forward movement only occurred during exhalation:

You should move forward during both inhalation and exhalation. Currently, you stand still while inhaling and move forward while exhaling. Right now, it feels more like a stop-and-go. It would be better in a continuous flow.

The visual feedback based on breathing, such as the movement of dots and squares, was also appreciated. Certain participants found it unpleasant that a predetermined breathing pace was enforced, whereas others indicated a preference for clearer indications of the breathing pace. In the end, we made the decision to not include breathing exercises in the IMH app. We selected 2 exercises that have also been demonstrated to be effective in reducing stress: progressive muscle relaxation and mindfulness [36,37].

#### Implementation

##### Technical Aspects

Several issues were encountered with the controllers. Some participants observed the controllers persistently on the screen, while others struggled with their operation. Participants found it favorable when minimal controller use was required:

Convenient and pleasant that you don’t have to use the controller system all the time, ensuring the focus is on relaxation.

There were also occasional challenges with setting up the play area’s boundaries. The IMH app uses smartphone-based VR headsets, which eliminate the need for a defined play area and do not require controllers. Furthermore, participants sporadically noted slight blurriness at the environment edges, an issue preventable by properly inserting the smartphone into the VR headset. In both the apps, it was also noted that errors in the environment (eg, occasional collisions with a rock or the campfire appearing almost on one’s lap) are detrimental to the perceived realism of the environment, bringing individuals back to the realization that they are not in a real world:

Sometimes, the plants move through a stone or through the fire, and it brings you back to the awareness that you are not completely in a real environment, but in a virtual one.

In response, the development of the IMH app focused on achieving the most realistic computer-generated environment feasible, while considering the technical constraints.

##### Cybersickness

In Flowborne, some participants experienced nausea due to a discrepancy between the movement in the VR headset and their actual physical movements. A participant expressed dissatisfaction in the following manner:

After the test, I felt sick and had a headache, which had an unpleasant impact on my body. To cope with the nausea and dizziness, I began to breathe less through my belly, aiming to move forward at a slower pace.

This phenomenon, known as cybersickness in the literature, results from an inconsistency between the sensory inputs [11]. The design of the IMH app ensures that there is no forward motion within the VR environment while the user is seated in a chair in reality, thereby reducing the risk of cybersickness within the limits of the processing power of a smartphone (ie, Android operated).

##### Acceptability and Usability

Some participants found themselves distracted by background noises, leading 1 individual to propose the use of headphones. Moreover, 1 individual questioned whether the app offers any added value compared to non-VR relaxation apps:

I’m not sure if this app offers a significant advantage over regular apps that indicate the rhythm of breathing through figures or sounds.

In addition, 1 person suggested to use the app in a standing position. Yet another participant described the environment as somewhat abandoned. Another individual expressed a willingness to use Flowborne again.

#### Experience

Certain participants reported feeling more relaxed after having used the VR relaxation apps. Overall, participants found the experience pleasant. However, responses did vary, with some participants reporting feelings of tiredness, others experiencing a sense of competitiveness, and some feeling energized, while 1 participant found the experience boring. Despite these diverse reactions, it is important to note that the primary aim of the IMH app is to facilitate relaxation for its users.

## Discussion

### Principal Findings

This study investigated users’ opinions on 2 existing stand-alone VR relaxation apps and demonstrated how, based on those insights, a new smartphone-based VR relaxation app was developed. The aim of this new app is to overcome the barriers that currently limit the use of VR for relaxation, thereby making VR relaxation accessible to a wider audience. These barriers include insufficient knowledge regarding VR operation, lack of availability of VR relaxation apps in the appropriate language for local health care systems, and cost concerns.

The evaluative study revealed 5 predominant themes that consistently recurred in the participants’ evaluations: audio, visuals, features, implementation, and experience. We evaluated the main findings of the participants for each theme and incorporated them into the development process of our new IMH app. This app is a Dutch-language, smartphone-based VR relaxation tool and is freely available for both Android [38] and iOS [39]. It is designed to enable users to perform relaxation exercises within a computer-generated nature setting, accompanied by natural and ethereal auditory backgrounds. The app has been developed to incorporate a high degree of variability and dynamic features within these environments. Moreover, the design ensures minimal cybersickness by eliminating movement within the virtual environment while the user remains physically still. The IMH app has multiple customization options, as research has shown that they can enhance relaxation levels [33]. Primarily, users have the option to select from 3 exercises: 1 free exploration without auditory guidance and 2 auditory-guided exercises, involving mindfulness or progressive muscle relaxation. Our decision to offer “free exploration” as an option aims to introduce users to the VR environment, while the choice of progressive muscle relaxation and mindfulness is based on scientific evidence demonstrating their effectiveness in stress reduction [36,37]. Second, users can choose between a male or female guiding voice for the mindfulness and progressive muscle relaxation exercises. The decision to incorporate a guiding voice stemmed from participants’ desire for clear instructions during the exercises. A third customization option allows users to select from 3 distinct environments: a tropical beach, a winter landscape featuring the northern lights, or a mountainous scenery.

### Comparison With Other Smartphone-Based VR Relaxation Apps

To the best of our knowledge, there exist a few other smartphone-based VR relaxation apps, although they are relatively scarce. One such app is the VR-based relaxation self-training app, which focuses on self-training for relaxation using diaphragmatic breathing and progressive muscle relaxation [40]. Users initiate their training by learning relaxation techniques in a computer-generated clinic, subsequently reviewing these techniques in a comfortable environment (eg, on a beach) and finally practicing in various computer-generated settings (eg, in an elevator). Researchers have also conducted an effectiveness study comparing this app with traditional learning methods using text and illustrations, finding that the VR app is particularly effective for progressive muscle relaxation exercises, with significantly lower tension levels observed compared to a control group using worksheets. Serenity is another gamified smartphone-based VR app developed to reduce stress among university students [41]. It features a traveler role-playing game format where the user visits various vacation spots under different settings, including day and night. The game allows players to trigger special events by collecting items along paths, with these items being selectable through eye-tracking technology. The effectiveness of this immersive experience was monitored through physiological sensors, with positive outcomes particularly among students with lower stress levels. Third, Gaia VR merges neurofeedback with biofeedback in a meditation app, using feedback from a heart rate band and an electroencephalogram headband to tailor the VR experience based on the user’s measured stress levels [42]. The user continuously wears the heart rate monitoring band throughout the day, enabling the app to suggest appropriate meditation techniques in response to the monitored stress levels. During the meditation sessions in VR, users can influence the virtual environment through their meditation-induced brainwave patterns, creating a dynamic and personalized meditation experience. This app has been tested thus far as a proof of concept; however, a more comprehensive evaluation is required to determine its long-term effectiveness and its applicability to a broader audience.

In summary, these 3 smartphone-based VR relaxation apps feature unique approaches and designs, differing from each other and from our IMH app, thereby demonstrating the extensive range of options available for inducing relaxation via smartphone-based VR. A key feature of the IMH app is its strong emphasis on both accessibility and customizability. As implied by its name, the Virtual Reality-Based Relaxation Self-Training app focuses mainly on the training of relaxation techniques. In contrast, the Serenity app targets students and uses gamification for stress reduction. The Gaia VR app primarily focuses on the integration of neurofeedback and biofeedback, providing real-time adjustments based on the user’s physiological state. Unlike the other 3 apps, the IMH app was developed specifically using user feedback. Additional research is needed to determine the most effective design for relaxation and to identify user preferences in smartphone-based VR relaxation.

### Comparison With Previous User Experience Studies

Although the effectiveness of VR for relaxation has been demonstrated in controlled research settings, insights into user experiences and preferred characteristics of VR relaxation apps remain sparse. It is important to acknowledge that, as far as we know, no user feedback studies specifically address smartphone-based VR relaxation. On the basis of 2 studies focusing on user experiences with VR for relaxation, although not specifically on smartphone-based VR, several aspects that users value can be highlighted [19,43]. Primarily, a study focusing explicitly on mindfulness VR demonstrated that sense of presence is a key factor [43]. Presence, which refers to the feeling of “being there” in a simulated environment, creates an “illusion of reality” where users behave as if the environment were real, despite being computer generated. Participants reported that this sense of presence within this virtual setting facilitated their mindfulness practices. Moreover, users noted that interruptions within the virtual environment could be distracting, leading to a reduction in the quality of the relaxation experience.

Second, users appreciated the integration of game elements into the VR relaxation experience. Features such as performance badges, use metrics, and leaderboards are appreciated and can therefore enhance the overall user experience and increase engagement. Nonetheless, the extensive diversity in the design and implementation of these gamification elements in different apps complicates the process of determining which elements are most preferred.

Third, the potential for personalizing the experience is a key factor for users. This includes choosing the computer-generated setting, adjusting the relaxation exercises provided, and selecting the background music, all contributing to a personalized and potentially more efficacious user experience.

Fourth, participants indicated that technical aspects, such as video resolution and the weight of the VR headset, are sometimes perceived as disruptive. They specifically highlighted that issues with blurred images and areas of low resolution within the video frequently led to distractions and adversely affected their experience. In summary, the 2 studies showed that users generally have a positive opinion of VR relaxation apps [19,43]. The findings demonstrated that VR relaxation app users value an experience that seems real, has gamification elements, can be customized, and runs well without glitches. These findings are in line with what participants mentioned in our evaluative study.

In developing the IMH app, we aimed to align closely with these preferences, although gamification elements were not integrated into our app. Another interesting study developed a framework for designing a virtual environment for stress therapy [44], listing 5 factors (ie, attention, attraction, environment setting, environment exploration, and interaction) containing 14 design elements: fascination stimuli, nature‐based theme, supporting audio, daylight lighting, realism, free exploration, predefined viewpoints, predefined path, passive interaction, active interaction, safety features, mind preparation, social support, and visual clarity. The IMH app meets 11 out of the 14 design elements, only lacking predefined paths, active interaction, or social support.

### Limitations

Several limitations of this study warrant attention. First, obtaining insights from professionals highly experienced in conducting relaxation sessions would have added depth. Second, our participant group was relatively young, which could hinder the generalization of our results to the general population. Therefore, it would be valuable to examine the opinions of older adults concerning VR relaxation apps as well. Third, it would have been intriguing to involve the participants not only in the evaluative study but also in the development process of the new app [45]. A systematic review on cocreating digital mental health interventions indicated that involving various stakeholders, such as health care professionals and patients, in the development process contributes to the creation of culturally sensitive and widely accepted interventions [46]. Fourth, our evaluation study focused on 2 stand-alone VR relaxation apps, rather than smartphone-based VR relaxation apps. It would have been interesting to obtain user feedback specifically on smartphone-based VR relaxation apps. Finally, concerning the methodology, coding iterations and the corresponding changes and evolution of the codes were not recorded, and discussions among the 3 coders about codes and themes were also not documented. Therefore, no interrater reliability could be calculated to assess the consistency of the different researchers’ coding. In this respect, it is not possible to quantitatively assess the validity and generalizability of the thematic analysis. However, we have described the process of thematic analysis in detail to ensure openness about the process.

### Strengths

We developed a new smartphone-based app, grounded in both an evaluative study and a review of the literature. We believe this approach substantiates a robust methodology for app development. Furthermore, to the best of our knowledge, there are few smartphone-based VR relaxation apps available, suggesting that our development has addressed a significant gap in the field. Moreover, we hypothesize that our IMH app could also be used as a method or tool to teach relaxation techniques. For instance, individuals may, after a few sessions of VR relaxation involving progressive muscle relaxation, potentially practice these techniques without VR.

### Future Directions

First, it is important to note that the presented app design, based on the assessed user preferences, has not yet been validated. This app design is an example of what a smartphone VR app for relaxation purposes could look like. In addition, future research should also focus on the efficacy and effectiveness of smartphone-based VR for relaxation. We have deliberately chosen to develop an app specifically for smartphone-based VR headsets to make VR relaxation accessible to a wider audience. Despite being more affordable and user-friendly than high-cost stand-alone VR systems, smartphone-based VR has its limitations, including a narrower field of view, lower resolution and frame rate, and less sophisticated optics and positional tracking. Nonetheless, a comparative study has not demonstrated significant differences in spatial presence, usability, cybersickness, satisfaction, workload, and learning outcomes between stand-alone VR headsets and smartphone-based VR in educational contexts [47]. Despite its limitations, smartphone-based VR might therefore still be able to deliver a satisfactory level of immersion [48]. To the best of our knowledge, no specific study has yet been conducted to explore the differences between smartphone-based VR and stand-alone VR specifically for relaxation purposes.

Second, additional investigation is required to examine other potentially engaging environments apart from natural settings, as well as to determine which particular aspects of nature are optimal for VR relaxation. In the design of the IMH app, the decision was made to include 3 different natural environments as options. This choice was driven by evidence from review studies showing that VR exposure to natural settings may lead to reductions in stress levels [34,49]. However, there is uncertainty in the literature regarding which type of VR environment is most effective for relaxation. Some researchers argue that the research has not adequately examined the diverse array of environments that can be used to promote relaxation in VR [50]. Besides urban computer-generated settings, only a handful of nonnatural alternatives have been extensively examined for VR relaxation. Consequently, a study was conducted to determine whether abstract environments could also serve as viable settings for VR relaxation. Results indicated that the effects on perceived restoration, including sensations of being away, experiencing soft fascination, and sustaining interest, were comparable across both abstract and natural environments, pointing to a similar restorative capacity in the abstract computer-generated environment [50]. According to another review, there is a lack of understanding regarding the differentiation between green and blue virtual natural environments [51]. In addition, research has been conducted on the impact of biodiversity on stress recovery within VR settings [52]. Researchers found that stress recovery was most effective in VR environments characterized by low biodiversity. Overall, further research is needed to explore alternative engaging environments beyond natural settings and to identify the specific elements of nature that are most effective for VR relaxation.

Third, further research is needed to better understand the potential advantages of interactivity in VR relaxation, particularly within the context of smartphone-based VR. Within the literature, there is debate regarding the role of interactivity in VR relaxation environments. The underlying principle of such interactivity involves engaging the user in specific tasks within the computer-generated environment, such as gaming or art creation, which are aimed at providing distraction and subsequent stress relief [53]. It is also suggested that interactive VR serves as an interesting and motivating tool, enhancing engagement among participants [54]. A review paper concluded that the effectiveness of interactivity in VR relaxation depends on the type of game used; interactive may surpass noninteractive VR relaxation in certain contexts [51]. The IMH app was designed without interactive features, partly because the typical constraints of smartphone-based VR, such as the lack of physical controllers, make interactivity less straightforward. Notably, feedback from our participants during evaluations indicated a preference for the absence of controllers, suggesting that this feature increases the usability of VR relaxation, thereby reducing barriers to adoption. This reflects the necessity of balancing considerations such as the potential advantages of interactivity with the ease provided by nonuse of controllers, demonstrating the importance of tailoring VR relaxation to individual preferences.

Further research is needed to explore the advantages of interactive features in VR relaxation, especially within smartphone-based VR apps. Exploring other mechanisms for interaction in smartphone-based VR, aside from controllers, such as head-based movements and voice control, could also be considered. Currently, our IMH app does not incorporate biofeedback, but integrating such a feature in the future could offer substantial advantages. Interventions that include biofeedback sensors with real-time monitoring of, for example, respiration, neural activity, heart rate, and skin conductance represent a promising advancement. This biological information can be communicated to the user via dynamic environmental changes, including alterations in music, sound, color, lighting, the appearance of objects, and animations, to facilitate the detection and regulation of internal states [50]. The combination of VR with biofeedback presents multiple benefits. For instance, VR can improve the visual appeal and salience of the feedback stimuli, which may, in turn, foster greater motivation and engagement. In our evaluative study, some participants also expressed appreciation for the visual feedback based on their breathing, such as the movement of dots and squares. In addition, biofeedback may enhance trainees’ belief in their capacity to relax both their body and mind, given that achievements in displaying healthy target behaviors or skills are instantly fed back and visible. Nevertheless, a significant obstacle is that most implementations of VR biofeedback demand considerable expertise and effort. Expensive physiological sensors must be purchased and integrated into the VR system [29]. Despite these challenges, it remains an intriguing avenue to explore. It would be interesting, for example, to develop a smartphone-based VR relaxation app that is linked to a smartwatch, which would provide feedback to the app.

### Conclusions

In this study, a new smartphone-based VR relaxation app was designed to address the current challenges in implementing VR relaxation technologies. The design of this app draws from an evaluation study on 2 existing stand-alone VR relaxation apps, supplemented by an extensive literature review to determine key attributes of VR relaxation apps. The resulting IMH app is a Dutch-language, smartphone-based VR app featuring a variety of customization options, namely, 3 different relaxation exercises, options for male or female guiding voices, and 3 unique environments. The environments are designed to be dynamic and varied and are accompanied by soothing music and natural sounds. The app design itself was nevertheless not validated in this study. Therefore, future research should validate the resulting design and explore the effectiveness of this app, along with a broader evaluation of its efficacy and user experience, to further build on the potential of VR relaxation apps.

## Abbreviations

IMH: Immersive Mental Health
VR: virtual reality

## Data Availability

The datasets generated and analyzed during this study are available from the corresponding author on reasonable request.
